# Supplementary material for: Identification of photoperiod-induced specific miRNAs in the adrenal glands of Sunite sheep (Ovis aries)
Source: Front Vet Sci. 2022 Jul 22;9:888207. doi: 10.3389/fvets.2022.888207 (PMC9354845; doi:10.3389/fvets.2022.888207)
Supplement: Supplementary file 1 [file Data_Sheet_1.ZIP › Supplementary file/miRDeep2 file.zip!/miRDeep2 file/Novel_Structure/SP42A1/Novel_307.pdf]

Provisional ID	:	NC_019467.2_13726
Score total	:	1.2
Score for star read(s)	:	-1.3
Score for read counts	:	0
Score for mfe	:	0.9
Score for randfold	:	1.6
Score for cons. seed	:	
Total read count	:	24
Mature read count	:	24
Loop read count	:	0
Star read count	:	0

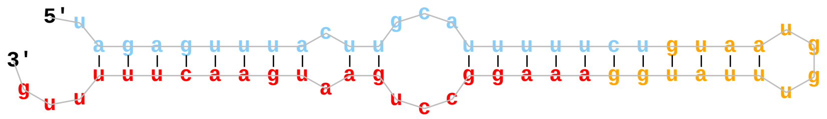

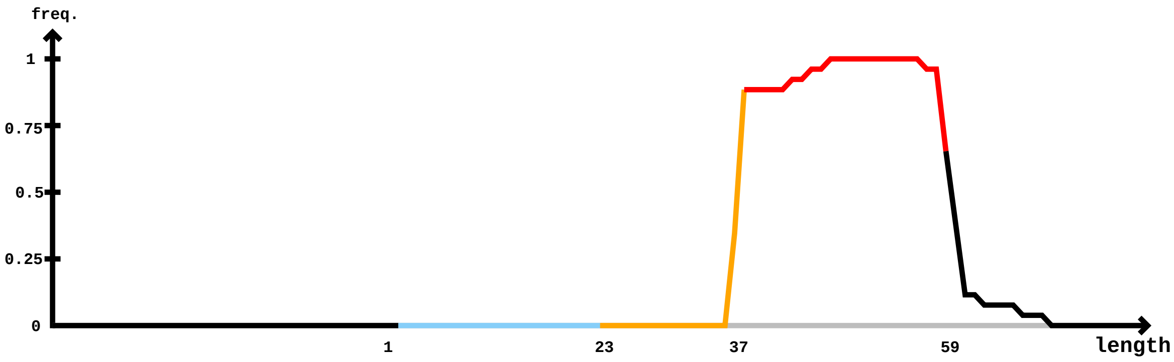

Star Mature

```
5'- aauugggcaauaaauuaaaauauugaguuggccuuagaguuuacuugcauuuuucuguaaugguuuauggaaaggccugaaugaacuuuuuggccaacccaauacaaagugg  -3'   exp
    .(((((.(((((........))))).(((((((..((((((((.((...(((((((((((....)))))))))))...)).))))))))..)))))))))))).........   reads    mm    sample
    .....................................................................gaaaggccugaaugaacuuuuu.....................   6        0     seq
    .....................................................................gaaaggccugaaugaacuuuuug....................   3        0     seq
    ......................................................................aaaggccugaaugaacuuu.......................   1        0     seq
    ......................................................................aaaggccugaaugaacuuuuu.....................   2        0     seq
    ......................................................................aaagAccugaaugaacuuuuug....................   1        1     seq
    ......................................................................aaaggccugaaugaacuuuuug....................   10       0     seq
    ...........................................................................ccugaaugaacuuuuuggcc.................   1        0     seq
    .............................................................................ugaaugaacuuuuuggccCacc.............   1        1     seq
    ...............................................................................aaugCacuuuuuggccaacccaa..........   1        1     seq
```
